# Supplementary material for: Mechanisms of life cycle simplification in African trypanosomes
Source: Nat Commun. 2024 Dec 2;15:10485. doi: 10.1038/s41467-024-54555-w (PMC11612274; doi:10.1038/s41467-024-54555-w)
Supplement: Supplementary file 1 — Supplementary information [file 41467_2024_54555_MOESM1_ESM.pdf]

Supplementary Materials for

Mechanisms of life cycle simplification in African trypanosomes

Guy R Oldrieve, Frank Venter, Mathieu Cayla, Mylène Verney, Laurent Hébert,
Manon Geerts, Nick Van Reet, Keith R Matthews
Corresponding author: guy.oldrieve@ed.ac.uk; keith.matthews@ed.ac.uk ;

The PDF file includes:

Figs. S1 to S6 and associated legends

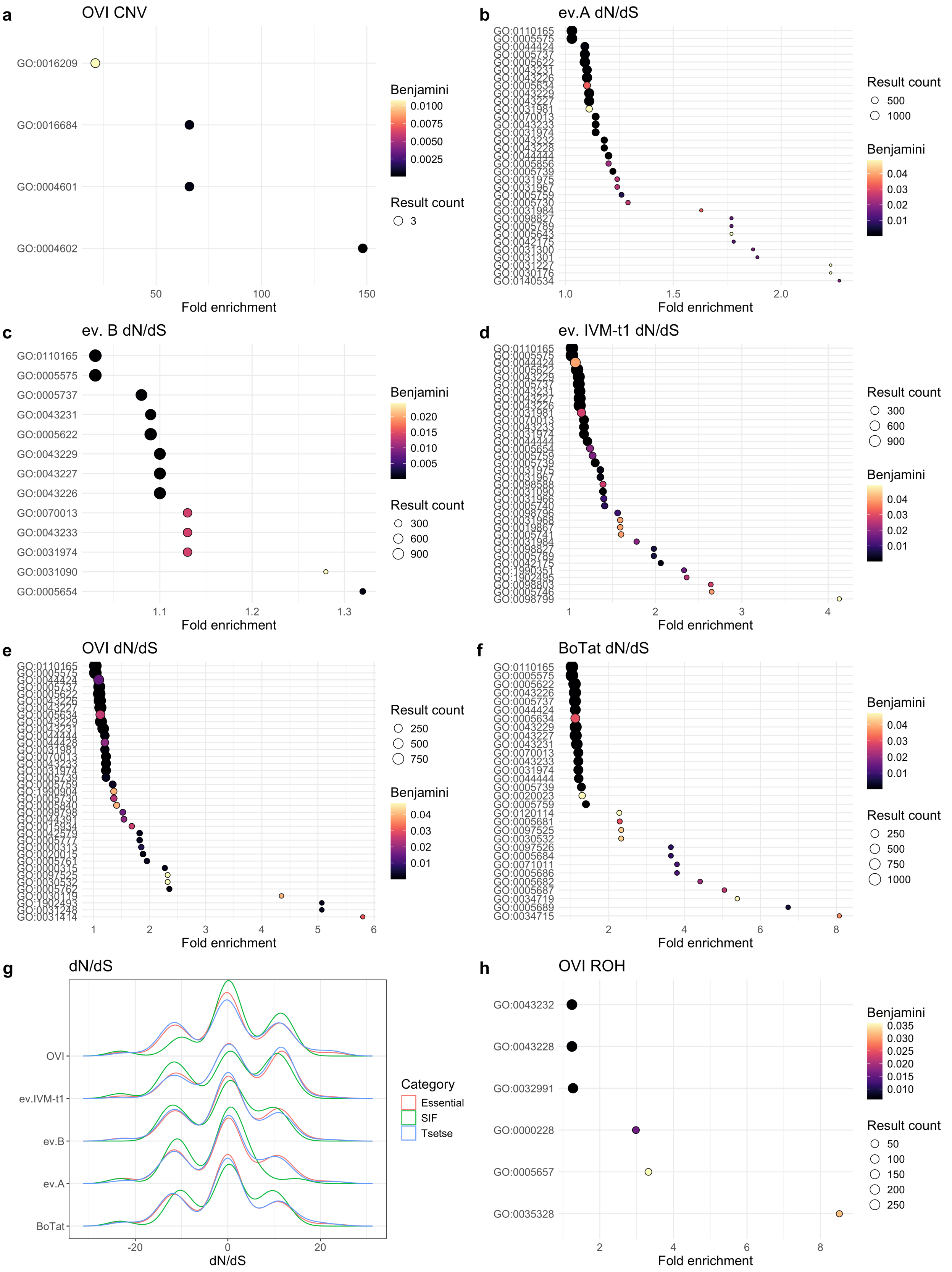

a
OVI CNV
GO:0016209
GO:0016684
GO:0004601
GO:0004602
50
100
150
Fold enrichment
Benjamini
0.0100
0.0075
0.0050
0.0025
Result count
3
b
ev.A dN/dS
1.0
1.5
2.0
Fold enrichment
Result count
500
1000
Benjamini
0.04
0.03
0.02
0.01
c
ev. B dN/dS
GO:0110165
GO:0005575
GO:0005737
GO:0043231
GO:0005622
GO:0043229
GO:0043227
GO:0043226
GO:0070013
GO:0043233
GO:0031974
GO:0031090
GO:0005654
1.1
1.2
1.3
Fold enrichment
Benjamini
0.020
0.015
0.010
0.005
Result count
300
600
900
d
ev. IVM-t1 dN/dS
1
2
3
4
Fold enrichment
Result count
300
600
900
Benjamini
0.04
0.03
0.02
0.01
e
OVI dN/dS
1
2
3
4
5
6
Fold enrichment
Result count
250
500
750
Benjamini
0.04
0.03
0.02
0.01
f
BoTat dN/dS
2
4
6
8
Fold enrichment
Benjamini
0.04
0.03
0.02
0.01
Result count
250
500
750
1000
g
dN/dS
OVI
ev.IVM-t1
ev.B
ev.A
BoTat
-20
0
20
dN/dS
Category
Essential
SIF
Tsetse
h
OVI ROH
GO:0043232
GO:0043228
GO:0032991
GO:0000228
GO:0005657
GO:0035328
2
4
6
8
Fold enrichment
Benjamini
0.035
0.030
0.025
0.020
0.015
0.010
Result count
50
100
150
200
250

Fig. S1.

Gene ontology enrichment. (A) *T. b. equiperdum* type OVI CNV 'glutathione peroxidase activity' (GO:0004602), 'peroxidase activity' (GO:0004601), 'oxidoreductase activity acting on peroxide as acceptor' (GO:0016684) and 'antioxidant activity' (GO:0016209). **(B)** *T. b. evansi* type A dN/dS; mitochondrion (GO:0005739) and mitochondrial matrix (GO:0005759), **(C)** *T. b. evansi* type B dN/dS; 'membrane-bounded organelle' (GO:0043227), 'intracellular membrane-bounded organelle' (GO:0043231), 'membrane-enclosed lumen' (GO:0031974) and 'organelle membrane' (GO:0031090**. (D)** *T. b. evansi* type IVM-t1 dN/dS; mitochondrion (GO:0005739), mitochondrial envelope (GO:0005740), mitochondrial membrane (GO:0031966), mitochondrial matrix (GO:0005759), mitochondrial outer membrane (GO:0005741), mitochondrial respirasome (GO:0005746), outer mitochondrial membrane protein complex (GO:0098799). **(E)** *T. b. equiperdum* type OVI dN/dS; mitochondrial large ribosomal subunit (GO:0005762), mitochondrion (GO:0005739), mitochondrial matrix (GO:0005759), mitochondrial ribosome (GO:0005761), mitochondrial protein-containing complex (GO:0098798). **(F)** *T. b. equiperdum* type BoTat dN/dS; mitochondrion (GO:0005739), mitochondrial matrix (GO:0005759). **(G)** dN/dS ratio variation for genes associated with the tsetse fly stage, QS pathway and essential genes, defined by phenotypes in each of the four RITseq libraries; monomorphic *T. brucei* have clade-specific variation in the efficacy of selection. **(H)** *T. b. equiperdum* type OVI ROH 'transcriptionally silent chromatin' (GO:0035328), 'replication fork' (GO:0005657) and 'non-membrane bound organelle' (GO:0043228)**.**

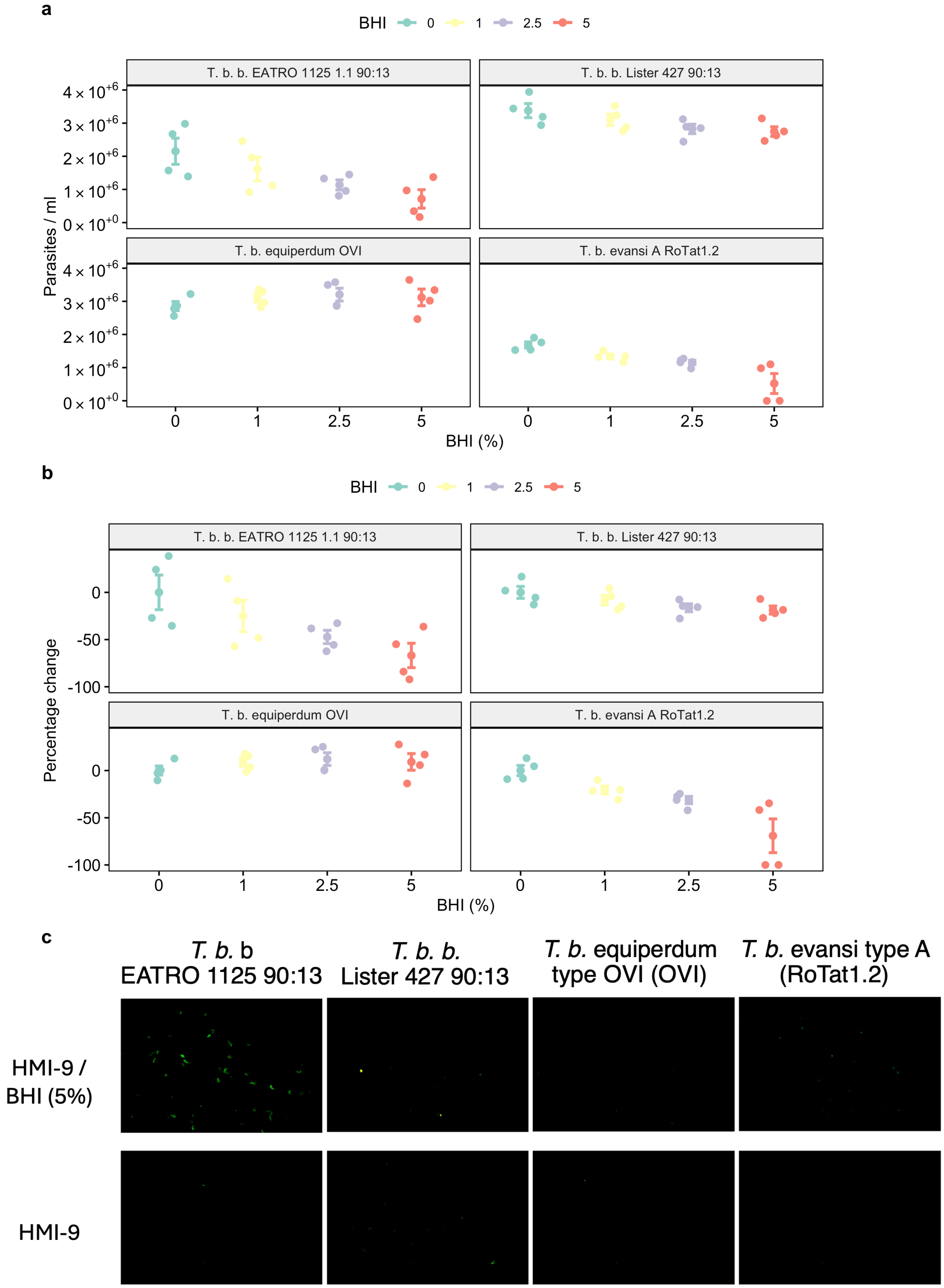
a
BHI 0 1 2.5 5
T. b. b. EATRO 1125 1.1 90:13
T. b. b. Lister 427 90:13
T. b. equiperdum OVI
T. b. evansi A RoTat1.2
Parasites / ml
BHI (%)
b
Percentage change
c
T. b. b EATRO 1125 90:13
T. b. b. Lister 427 90:13
T. b. equiperdum type OVI (OVI)
T. b. evansi type A (RoTat1.2)
HMI-9 / BHI (5%)
HMI-9

45

Fig. S2.

Growth of *T. brucei* strains exposed to BHI supplemented media for 48 hours. Pleomorphic *T. b. brucei* EATRO 1124 90:13 and monomorphic *T. b.* Lister 427, *T. b. equiperdum* type OVI (OVI isolate) and *T. b. evansi* type A (Rotat1.2 isolate) (N = 4 replicates for each cell line) were exposed to BHI at either 0, 1, 2.5 or 5 percent. The data was compared as **(A)** parasite density or **(B)** percentage change, calculated as the density of each replicate at each BHI concentration compared to the mean density at 0% BHI for the corresponding strain. Error bars=mean standard error **(C)** Representative PAD1 images of each cell line grown in 0% and 5% BHI.

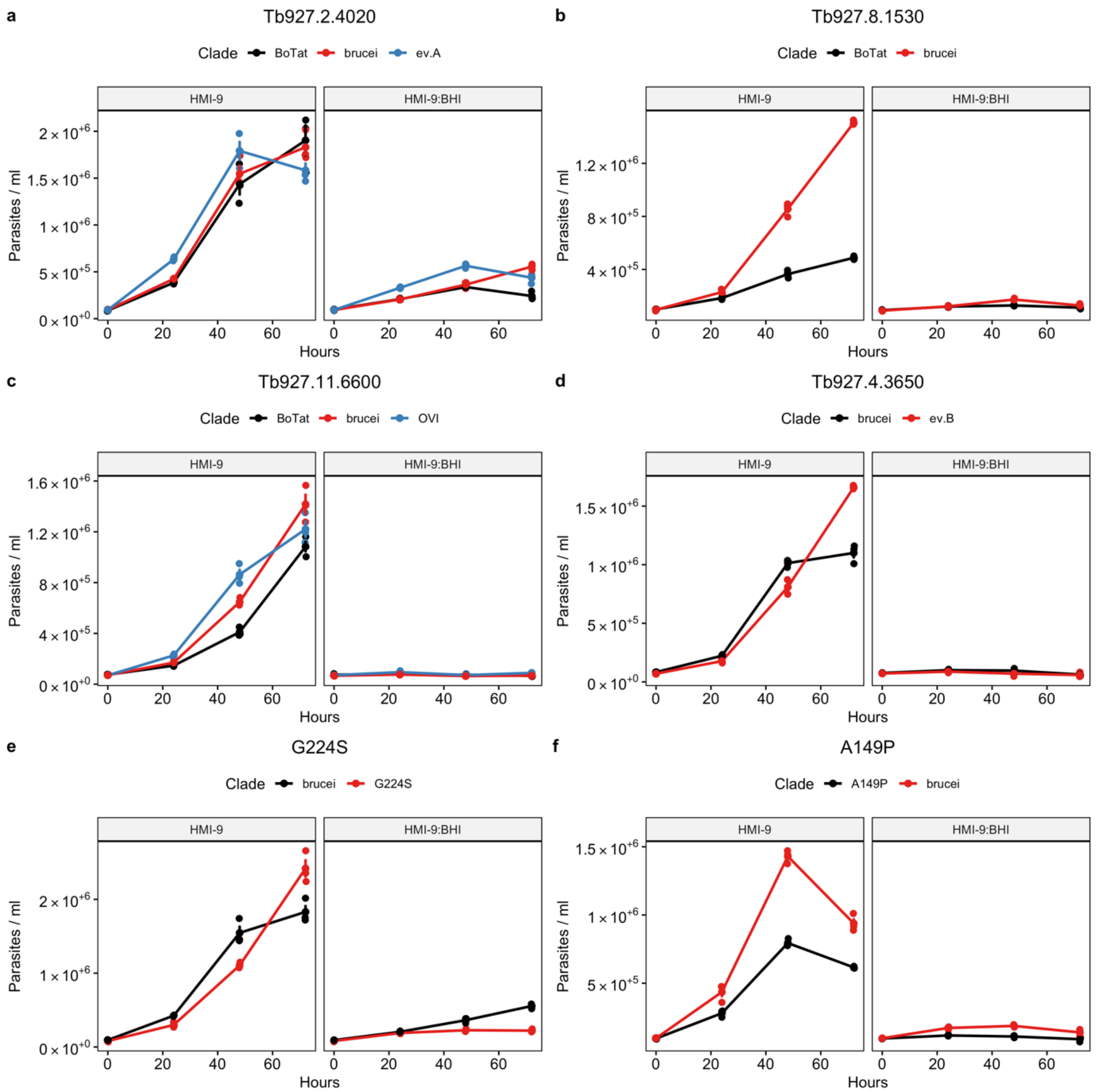

Fig. S3.

Growth of pleomorphic *T. brucei* EATRO 1125 AnTat1.1 subjected to replacement of endogenous gene targets. (A) Tb927.2.4020 APPBP1, **(B)** Tb927.8.1530 - Golgi pH regulator (GPR89), **(C)** Tb927.11.6600 – Hyp1, **(D)** Tb927.4.3650 – Protein phosphatase 1 (PP1), **(E)** G224S, **(F)** A149P. The replacement cell lines were grown in HMI-9, or HMI-9 supplemented with an in vitro

mimic of the QS signal, oligopeptide broth BHI (15%). Error bars=mean standard error and N = 3 replicates for each cell line.

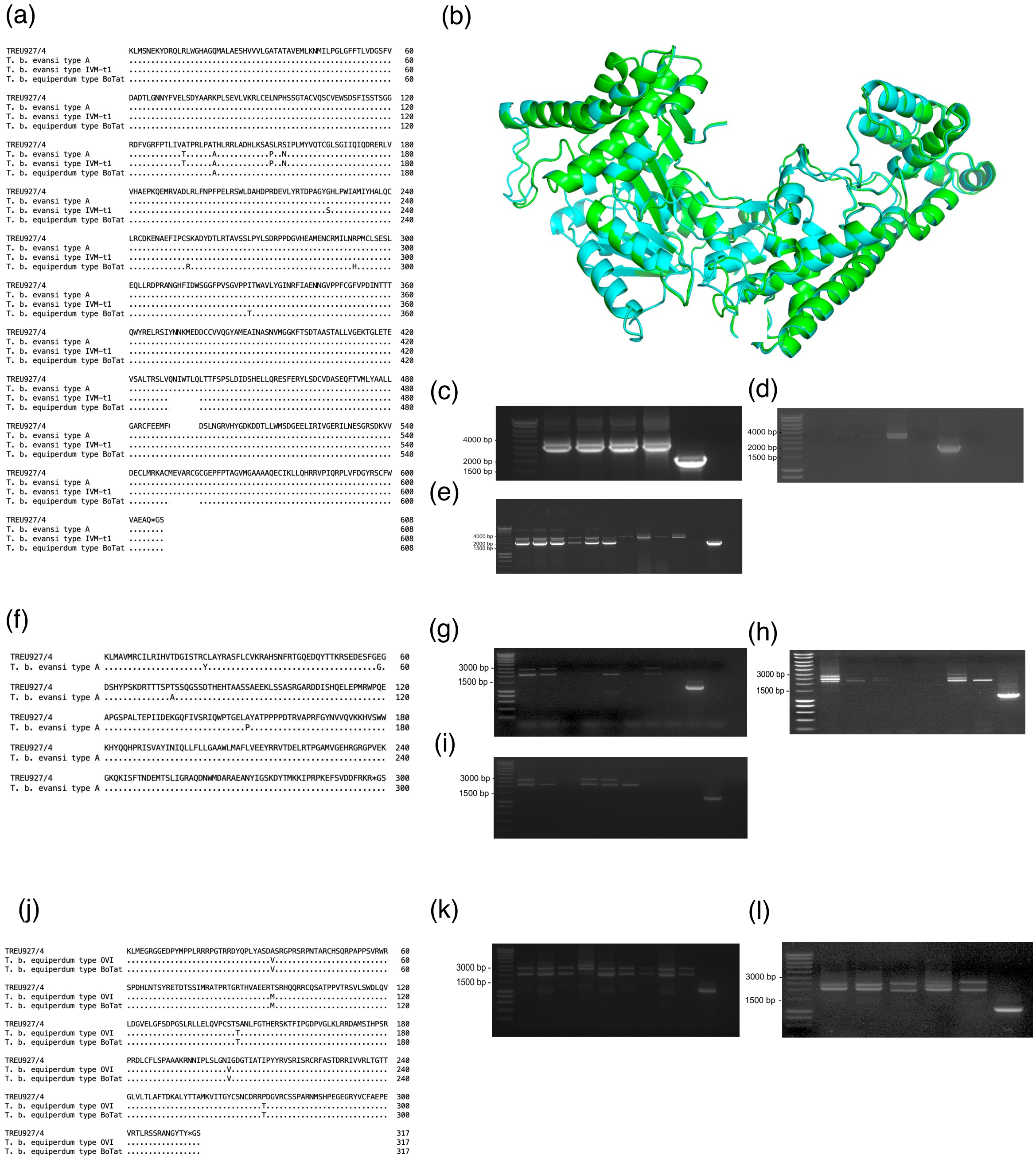

Fig. S4

Confirmation of replacement of endogenous gene targets in pleomorphic *T. brucei* EATRO 1125 AnTat1.1. Tb927.2.4020 **(A)** gene alignment **(B)** *T. b. brucei*

(blue) and *T. b. evansi* type IVM-t1 (green) protein structure alignment (RMSD = 0.210). **(C-E)** PCR confirmation of endogenous allele replacement. **(C)** Replacement of wild-type APPBP1 (Tb927.2.4020) with the *T. b. equiperdum* type BoTat sequence (clone 4, lane 1), *T. b. evansi* type A sequence (clone 1, lane 2), *T. b. evansi* type IVM-t1 sequence (clone 3, lane 3), *T. b. brucei* sequence (clone 1, lane 4), control J1339 (lane 5) and negative (lane 6). **(D)** Homozygous phleomycin+G418 add-back (clones 2-3, lanes 1-2), homozygous G418+phleomycin add-back (clones 2-3, lanes 3-4), negative (lane 5) and control J1339 (lane 6). **(E)** G224S heterozygous replacement (clones 1-6, lanes 1-6) and homozygous replacements (clones 1-4, lanes 7-10), negative (lane 11) and control J1339 (lane 12). Expected sizes (bp): Wild type Tb927.2.4020 = 1,992, pPOT-Hygromycin-Tb927.2.4020 = 3,425, pPOT-Blasticidin-Tb927.2.4020 = 2,996, pPOT-Phleomycin-Tb927.2.4020 = 2,221, pPOT-Neomycin-Tb927.2.4020 = 3,194. Tb927.5.2580 **(F)** gene alignment **(G-I)** PCR confirmation of endogenous allele replacement. **(G)** Replacement of wild-type Tb927.5.2580 with *T. b. evansi* type A sequence (clones 1-3, lanes 1-3) and the *T. b. brucei* sequence (lanes 4-8, clones 1-5) and control J1339 (lane 10). **(H)** Homozygous add-back (clones 1-7), control J1339 (lane 8), negative (lane 9). **(I)** A149P sequence (clones 1-7, lanes 1-7), negative (lane 9) and control J1339 (lane 10). Expected sizes (bp): Wild type Tb927.5.2580 = 1,134, pPOT-Hygromycin-Tb927.5.2580 = 2,337, pPOT-Blasticidin-Tb927.5.2580 = 1,908, pPOT-Phleomycin-Tb927.5.2580 = 1,893, pPOT-Neomycin-Tb927.5.2580 = 2,106. Tb927.11.3400 **(J)** gene alignment **(K-L)** PCR confirmation of endogenous allele replacement. **(K)** Replacement of wild-type Tb927.11.3400 *T. b. equiperdum* type BoTat (clones 1-3, lanes 1-3) sequence, *T. b. brucei* (clones 1-3, lanes 4-6) sequence and *T. b. equiperdum* type OVI (clones 1-3, lanes 7-9) sequence, control J1339 (lane 10) and negative (lane 11) **(L)** *T. b. equiperdum type OVI* homozygous add-back (clone 1-2, lanes 1-2), *T. b. equiperdum* type BoTat homozygous add-back (clone 1-2, lanes 3-4), *T. b. brucei* initial replacement control (lane 5), control J1339 (lane 6) and negative (lane 7). Expected sizes (bp): Wild type Tb927.11.3400 = 1,110, pPOT-Hygromycin-Tb927.11.3400 = 2,552 and pPOT-Blasticidin-Tb927.11.3400 = 2,123, pPOT-Phleomycin-

Tb927.11.3400 = 2,108, pPOT-Neomycin- Tb927.11.3400 = 2,321. Source data are provided as a Source Data file.

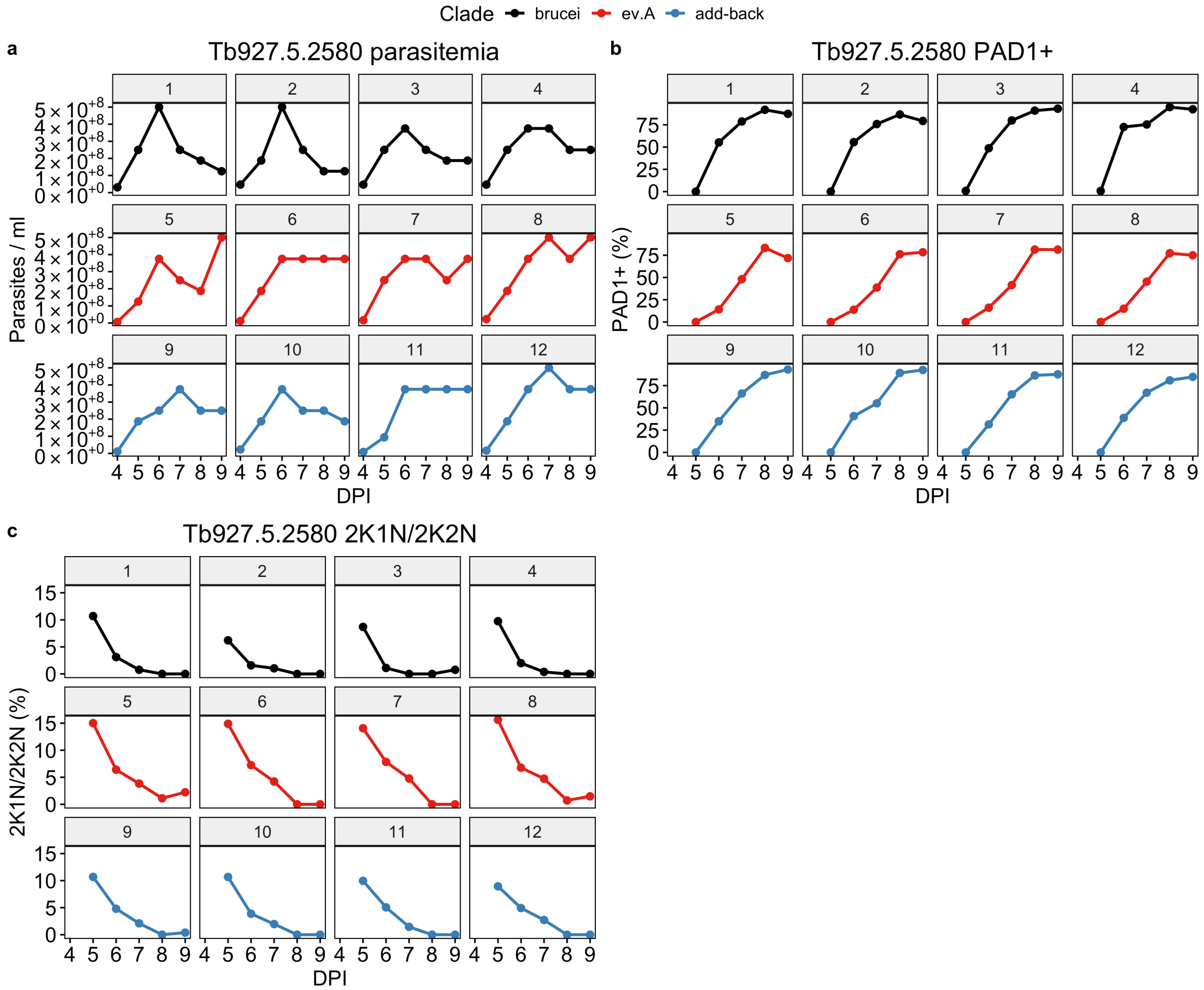

Figure S5

Pleomorphic *T. b. brucei* expressing the monomorphic *T. b. evansi* type A Tb927.5.2580 sequence delays developmental progression in vivo. (A) In vivo growth of pleomorphic *T. b. brucei* expressing the monomorphic *T. b. evansi* type A Tb927.5.2580 sequence or the pleomorphic *T. b. brucei* sequence, **(B)** cell cycle stage and **(C)** percentage of PAD1+ cells as assessed by immunofluorescence. Each cell line was used to infect four mice, represented by each panel.

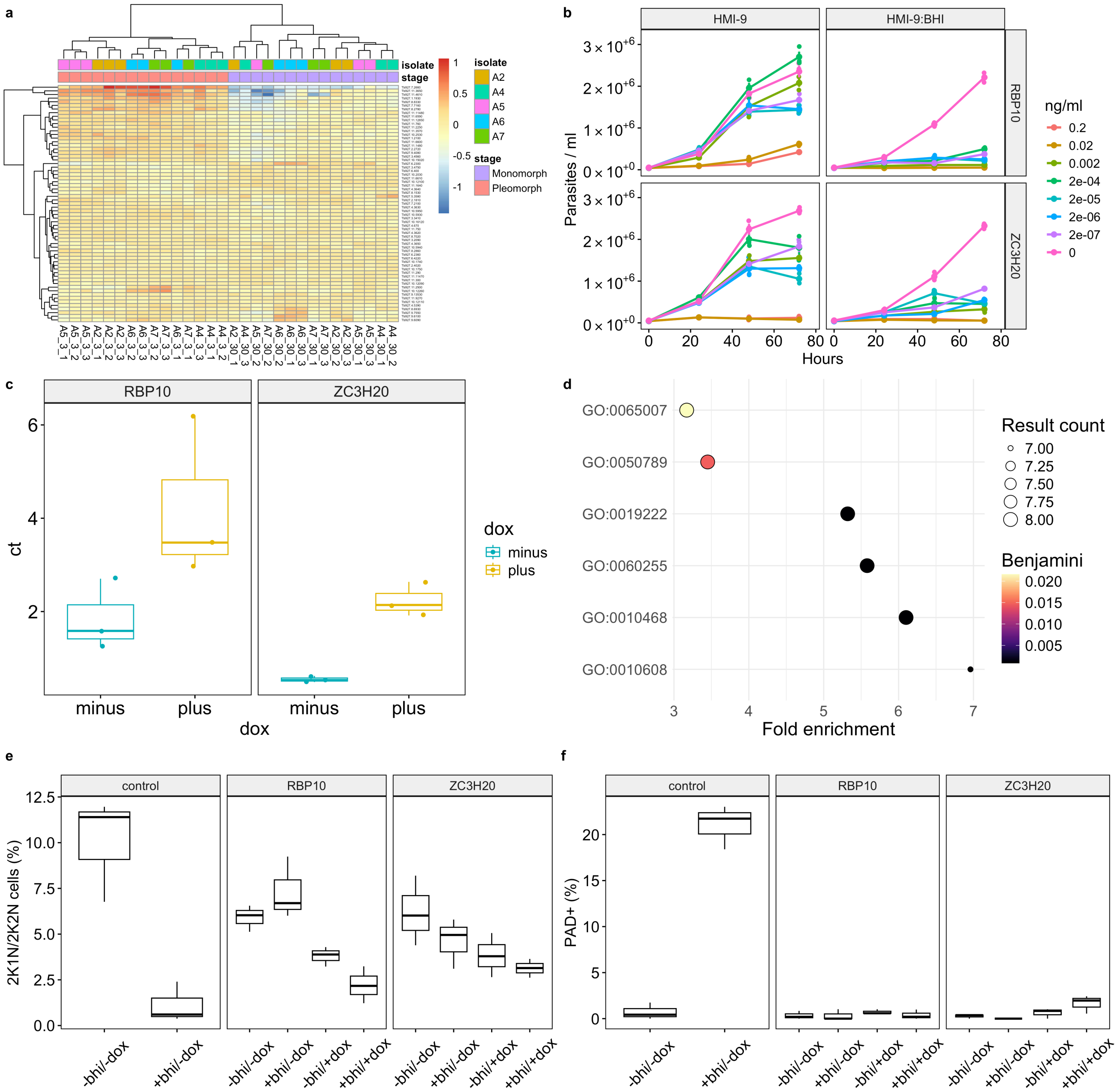

Figure S6

Selected monomorphs display reduced developmental progression, which is reversible by inducible expression of key gene regulators. **(A)** Heatmap of SIF genes. Normalisation was achieved by calculating the deviation of expression from each gene in a specific sample from the mean expression of that gene across all samples. Blocks of genes and samples which covary are clustered, indicated by branches. The split between each stage of the selection was not specified. **(B)** Growth of clone A7 in HMI-9 or HMI-9: BHI induced to overexpress RBP10 or

ZC3H20 at titrated concentrations of doxycycline; Error bars=mean standard error **(C)** Quantification was calculated via RT-qPCR using ZC3H20 or RBP10 specific primers and cDNA generated from clone A7 induced to overexpress either gene. The expression of the gene of interest was normalised to ZFP3 and compared between uninduced (0ng/ml) and induced (0.002ng/ml) cultures grown in triplicate (2^-(ΔΔCT))**. (D)** GO molecular function enrichment from 23 commonly differentially expressed transcripts included significant enrichment of ‘regulation of gene expression’ (GO:0010468), ‘posttranscriptional regulation of gene expression’ (GO:0010608) and ‘regulation of macromolecule metabolic processes’ (GO:0060255). **(E & F)** percentage of the population replicating and PAD1+% from the monomorphic clone A7 induced with doxycycline to express transgenic RBP10 and ZC3H20 or not and grown in HMI-9 or HMI-9 supplemented with 15% BHI. For panels C, D and E, boxplot = Minium, 25th percentile, Median, 75th percentile and Maximum (https://r-graph-gallery.com/boxplot.html). For panels B, C, D and E N=4 replicates.
