# Supplementary material for: Mechanisms of life cycle simplification in African trypanosomes
Source: Nat Commun. 2024 Dec 2;15:10485. doi: 10.1038/s41467-024-54555-w (PMC11612274; doi:10.1038/s41467-024-54555-w)
Supplement: Supplementary file 3 — Description Of Additional Supplementary File [file 41467_2024_54555_MOESM3_ESM.pdf]

Description of additional supplementary file

Supplementary data 1

Database of trypanosome isolates, sequencing metrics, region of origin and sequencing technology used to derive the genomic datasets in this study.

Supplementary data 2

Clade-specific variants were prioritised to create a target list of genes to validate their role in monomorphism. Initially, pseudogenes, VSGs and genes not on the megabase chromosomes 1-11 were removed. These genes were further filtered to create two target categories using the following criteria: Category 1 (Tab 1) Genes which have a clade specific high-impact or moderate impact mutation in a monomorphic clade in a known QS pathway gene. The gene must also display no high impact mutations in any pleomorphic isolates. Category 2 (Tab 2). Genes which have a clade specific high-impact or moderate impact mutation in a monomorphic clade along with a dN/dS ratio 1 in any monomorphic clade. The gene must also have a smaller log fold change in D3, D6 and PF than in the DIF category whilst displaying a log fold change in the DIF category greater than -1.5. The gene must also display no high impact mutations in any pleomorphic isolates. (Tab 3) genes with a dN/dS ratio >1 in all monomorphic clades and <=1 in the pleomorphic background.

Supplementary data 3

Oligonucleotide primer and antibody resources used in this study.

Supplementary movie 1

Cell motility video tracks for T. brucei expressing Tb927.11.3400 from wild type cells (T. b. brucei AnTat 1.1)

Supplementary movie 2

Cell motility video tracks for T. brucei expressing Tb927.11.3400 from T. b. equiperdum BoTat

Supplementary movie 3

Cell motility video tracks for T. brucei expressing Tb927.11.3400 from T. b. equiperdum BoTat with T. b. brucei AnTat1.1 sequence as add back

Supplementary movie 4

Cell motility video tracks for T. brucei expressing Tb927.11.3400 from T. b. equiperdum OVI

Supplementary movie 5

Cell motility video tracks for T. brucei expressing Tb927.11.3400 from T. b. equiperdum OVI with T. b. brucei AnTat1.1 sequence as add back
