# Supplementary material for: Mechanisms of life cycle simplification in African trypanosomes
Source: Nat Commun. 2024 Dec 2;15:10485. doi: 10.1038/s41467-024-54555-w (PMC11612274; doi:10.1038/s41467-024-54555-w)
Supplement: Supplementary file 12 — Reporting summary [file 41467_2024_54555_MOESM12_ESM.pdf]

nature portfolio

Corresponding author(s): Keith R Matthews
Guy Oldrieve

Last updated by author(s): 14-7-2024

Reporting Summary

Nature Portfolio wishes to improve the reproducibility of the work that we publish. This form provides structure for consistency and transparency in reporting. For further information on Nature Portfolio policies, see our Editorial Policies and the Editorial Policy Checklist.

Statistics

For all statistical analyses, confirm that the following items are present in the figure legend, table legend, main text, or Methods section.

n/a	Confirmed	
☐	☒	The exact sample size (*n*) for each experimental group/condition, given as a discrete number and unit of measurement
☐	☒	A statement on whether measurements were taken from distinct samples or whether the same sample was measured repeatedly
☐	☒	The statistical test(s) used AND whether they are one- or two-sided<br>*Only common tests should be described solely by name; describe more complex techniques in the Methods section.*
☒	☐	A description of all covariates tested
☐	☒	A description of any assumptions or corrections, such as tests of normality and adjustment for multiple comparisons
☐	☒	A full description of the statistical parameters including central tendency (e.g. means) or other basic estimates (e.g. regression coefficient) AND variation (e.g. standard deviation) or associated estimates of uncertainty (e.g. confidence intervals)
☐	☒	For null hypothesis testing, the test statistic (e.g. *F*, *t*, *r*) with confidence intervals, effect sizes, degrees of freedom and *P* value noted<br>*Give P values as exact values whenever suitable.*
☒	☐	For Bayesian analysis, information on the choice of priors and Markov chain Monte Carlo settings
☒	☐	For hierarchical and complex designs, identification of the appropriate level for tests and full reporting of outcomes
☒	☐	Estimates of effect sizes (e.g. Cohen's *d*, Pearson's *r*), indicating how they were calculated

Our web collection on statistics for biologists contains articles on many of the points above.

Software and code

Policy information about availability of computer code

Data collection	All code written for this study has been deposited on GitHub (https://github.com/goldrieve/Mechanisms-of-life-cycle-simplification). The workflow was implemented with SnakeMake.
Data analysis	The quality of the raw reads was analysed with FastQC and subjected to quality trimming with Trimmomatic. The trimmed reads were aligned to the T. brucei TREU927/4V5 reference genome with bwa-mem. The reads were prepared for variant calling following the GATK4 best practices pipeline, which included marking duplicate reads. The variants were combined and filtered with stringent cut-offs, in keeping with GATK's best practices pipeline and previous studies. The complete list of genomes analysed in this study, including their accession IDs, is summarised in Supplementary Data S1. Clade-specific mutations were assigned to annotated genes in the TRUE927/4 reference genome using snpEFF. Protein domains and putative phosphorylation sites were identified in target genes using InterProScan accessed through TriTrypDB (www.TritrypDB.org). snpSIFT was then used to identify mutations which were specific to a monomorphic clade using the case-control function . For detailed variant analysis, genome feature summaries and target prediction, full information is provided in the Methods.

For manuscripts utilizing custom algorithms or software that are central to the research but not yet described in published literature, software must be made available to editors and reviewers. We strongly encourage code deposition in a community repository (e.g. GitHub). See the Nature Portfolio guidelines for submitting code & software for further information.

Data

Policy information about availability of data

All manuscripts must include a data availability statement. This statement should provide the following information, where applicable:

- Accession codes, unique identifiers, or web links for publicly available datasets
- A description of any restrictions on data availability
- For clinical datasets or third party data, please ensure that the statement adheres to our policy

All data and code are available in the main text, the supplementary materials, GitHub (https://github.com/goldrieve/Mechanisms-of-life-cycle-simplification) or NCBI under the BioProject PRJNA1114649; reviewer access link :
https://eur02.safelinks.protection.outlook.com/?url=https%3A%2F%2Fdataview.ncbi.nlm.nih.gov%2Fobject%2FPRJNA1114649%3Freviewer%3Dvth6tbkquoagp9i2eloq8u1qdj&data=05%7C02%7C%7Cb9733b49fe834be075bd08dca5aafab5%7C2e9f06b016694589878910a06934dc61%7C0%7C0%7C638567400972824670%7CUnknown%7CTWFpbGZsb3d8eyJWIjoiMC4wLjAwMDAiLCJQIjoiV2luMzIiLCJBTiI6Ik1haWwiLCJXVCI6Mn0%3D%7C0%7C%7C%7C&sdata=wemaXX2i%2FqgDMiBbMKB5arI%2FRf86o6gmAV2hi%2FbS9pM%3D&reserved=0

Research involving human participants, their data, or biological material

Policy information about studies with human participants or human data. See also policy information about sex, gender (identity/presentation), and sexual orientation and race, ethnicity and racism.

Reporting on sex and gender	N/A
Reporting on race, ethnicity, or other socially relevant groupings	N/A
Population characteristics	N/A
Recruitment	N/A
Ethics oversight	N/A

Note that full information on the approval of the study protocol must also be provided in the manuscript.

Field-specific reporting

Please select the one below that is the best fit for your research. If you are not sure, read the appropriate sections before making your selection.

☒ Life sciences ☐ Behavioural & social sciences ☐ Ecological, evolutionary & environmental sciences

For a reference copy of the document with all sections, see nature.com/documents/nr-reporting-summary-flat.pdf

Life sciences study design

All studies must disclose on these points even when the disclosure is negative.

Sample size	For the analysis of phenotypes 3- 5 animals per treatment were routinely used for analysis. In previous analyses (e.g. Mony, B.M., et al., Genome-wide dissection of the quorum sensing signalling pathway in Trypanosoma brucei. Nature, 2014. 505(7485): p. 681-5) we showed that this sample size is sufficient to detect differences between cell lines and treatment groups (for example where gene silencing is activated by provision of doxycycline). In that study, we tested 5 genes for effects with and without doxycycline mediated gene-silencing in vivo. Using cell cycle status as the measured parameter, the effect size ranged from 0.637 to 1.804. Those values were then used to calculate the power for different samples sizes. This showed that a sample size of 3-5 per group (+ or - DOX) , or total of 6 to 10 allowed us to achieve 80% power for all test genes except one. In most cases observed phenotypes were detected in pilot studies in addition to the presented datasets.
Data exclusions	No data were excluded
Replication	All figures include information on the replicate number for each experiment. Experiments were also validated in additional pilot experiments or independent replicates.
Randomization	Animals were allocated at random into treatment groups from a group of female, age matched MF1 mice
Blinding	No blinding was carried out

Reporting for specific materials, systems and methods

We require information from authors about some types of materials, experimental systems and methods used in many studies. Here, indicate whether each material, system or method listed is relevant to your study. If you are not sure if a list item applies to your research, read the appropriate section before selecting a response.

Materials & experimental systems

n/a	Involved in the study
☐	☒ Antibodies
☐	☒ Eukaryotic cell lines
☒	☐ Palaeontology and archaeology
☐	☒ Animals and other organisms
☒	☐ Clinical data
☒	☐ Dual use research of concern
☒	☐ Plants

Methods

n/a	Involved in the study
☒	☐ ChIP-seq
☒	☐ Flow cytometry
☒	☐ MRI-based neuroimaging

Antibodies

Antibodies used

Anti-PAD1, Dean et al., 2009.
Anti-rabbit (goat anti-rabbit IgG (H+L) Dylight 800; Thermofisher Cat#SA5-10036
Anti-Ty1 epitope tag specific BB2 antibody. Bastin et al., 1996; hybridoma cell line a gift of Keith Gull, Oxford University/available through Thermofisher. Cat#MA5-23513; RRID:AB_2610644
Anti-Mouse AlexaFluor 568. abcam. Cat# ab175701

Validation

Anti-PAD1, Dean et al., 2009.
Anti-rabbit (goat anti-rabbit IgG (H+L) Dylight 800; Thermofisher Cat#SA5-10036
Anti-Ty1 epitope tag specific BB2 antibody. Bastin et al., 1996; hybridoma cell line a gift of Keith Gull, Oxford University/available through Thermofisher. Cat#MA5-23513; RRID:AB_2610644
Anti-Mouse AlexaFluor 568. abcam. Cat# ab175701

Eukaryotic cell lines

Policy information about cell lines and Sex and Gender in Research

Cell line source(s)

Trypanosoma brucei EATRO 1125 AnTat1.1 J1339; Rojas et al 2018; Genomic analyses were performed on genomic material from cell lines and isolates detailed in Supplementary Data 1

Authentication

Molecular validation (inducible expression of integrated target genes or successful gene knockout)

Mycoplasma contamination

Not tested

Commonly misidentified lines (See ICLAC register)

N/A

Animals and other research organisms

Policy information about studies involving animals; ARRIVE guidelines recommended for reporting animal research, and Sex and Gender in Research

Laboratory animals

Female age matched MF1 mice were used

Wild animals

N/A

Reporting on sex

Female mice were used throughout

Field-collected samples

N/A

Ethics oversight

All work was carried out under a UK home office licence (P262AE604) that had been approved after local ethical review.

Note that full information on the approval of the study protocol must also be provided in the manuscript.

Plants

Seed stocks	N/A
Novel plant genotypes	N/A
Authentication	N/A
